# Age-Related Changes in the Response of Finger Skin Blood Flow during a Braille Character Discrimination Task

**DOI:** 10.3390/healthcare9020143

**Published:** 2021-02-01

**Authors:** Jun Murata, Shin Murata, Takayuki Kodama, Hideki Nakano, Masayuki Soma, Hideyuki Nakae, Yousuke Satoh, Haruki Kogo, Naho Umeki

**Affiliations:** 1 Department of Physical and Occupational Therapy, Graduate School of Biomedical Sciences, Nagasaki University, Nagasaki 852-8520, Japan; kogoha@nisikyu-u.ac.jp (H.K.); naho.umeki@outlook.jp (N.U.); 2 Department of Physical Therapy, Faculty of Health Sciences, Kyoto Tachibana University, Kyoto 607-8175, Japan; murata-s@tachibana-u.ac.jp (S.M.); kodama-t@tachibana-u.ac.jp (T.K.); nakano.neuroreha@gmail.com (H.N.); 3 Course of Rehabilitation, Department of Health Sciences, Tohoku Fukushi University, Miyagi 981-8522, Japan; souma@tfu-mail.tfu.ac.jp (M.S.); hide-n@tfu-mail.tfu.ac.jp (H.N.); yosuke-s@tfu-mail.tfu.ac.jp (Y.S.)

**Keywords:** skin blood flow, tactile-pressure threshold, discrimination task, aging

## Abstract

We hypothesized that age-related changes in sensory function might be reflected by a modulation of the blood flow response associated with tactile sensation. The aim of the present study was to clarify how the blood flow response of the fingers during concentrated finger perception is affected by aging. We measured the tactile-pressure threshold of the distal palmar pad of the index finger and skin blood flow in the finger (SBF) during Braille reading performed under blind conditions in young (*n* = 27) and older (*n* = 37) subjects. As a result, the tactile-pressure threshold was higher in older subjects (2.99 ± 0.37 log_10_ 0.1 mg) than in young subjects (2.76 ± 0.24 log_10_ 0.1 mg) (*p* < 0.01). On the other hand, the SBF response was markedly smaller in older subjects (−4.9 ± 7.0%) than in young subjects (−25.8 ± 15.4%) (*p* < 0.01). Moreover, the peak response arrival times to Braille reading in older and young subjects were 12.5 ± 3.1 s and 8.8 ± 3.6 s, respectively (*p* < 0.01). A decline in tactile sensitivity occurs with aging. Blood flow responses associated with tactile sensation are also affected by aging, as represented by a decrease in blood flow and a delay in the reaction time.

## 1. Introduction

Age-related sensory impairment is slow and gradual progress that affects not only the main sensory modalities (vision, hearing, taste, and smell) but also somatosensory functions, including tactile sensitivity. Previous studies reported that the tactile-pressure threshold of the hands was higher in older subjects than in young subjects [1,2,3]. Tactile acuity deteriorates with age, and this decline is particularly prominent at the fingertip, at which the doubling and tripling of thresholds for the resolution of spatial stimuli suggest a marked reduction in functional tactile innervation [4,5,6]. Moreover, a decline with age in the tactile sensibility of the hands has been closely associated with the deterioration of manual function [7], resulting in worsening performance in the activities of daily living, such as fastening buttons, tying shoelaces, and writing a note [8].

On the other hand, the human hand is highly vascularized [9]. The glabrous skin areas of the hand are rich in arteriovenous anastomoses, which causes large fluctuations in skin blood flow (SBF) in these areas. We recently reported that blood flow in the hand was reduced via sympathetic nerve activity during a Braille character discrimination task [10,11]. Furthermore, Macefield and Elam [12,13] demonstrated that the firing of tactile afferents in the human finger pads was associated with arterial pulsation within the finger. These findings suggested that the regulation of blood flow to the fingers plays an important role in sensory mechanisms. However, aging is associated with changes in cardiovascular regulation as well as sensory functions. Thus, age-related changes in tactile sensibility may be reflected by the modulation of the blood flow response to concentrated finger perception. To examine this hypothesis, we measured the response of finger SBF during a Braille character discrimination task in older and young subjects and compared responses to the task between the two age groups.

## 2. Materials and Methods

### 2.1. Participants

Sixty-four volunteers (older: *n* = 37, 33 women and 4 men, mean = 71.7 years, range 64–79 years; young: *n* = 27, 24 women and 3 men, mean = 20.7 years, range 20–24 years) participated in the present study. All young subjects were healthy and had not taken any medication. Older subjects professed to be in good health on a standard medical examination questionnaire and lived independently in the community. None of the subjects had common diseases that are known to influence the neuromuscular function of the hand, such as carpal tunnel syndrome, osteoarthritis of the hand joints, diabetic polyneuropathy, or cervical spondylosis-related cervical radiculopathy. However, twenty-two subjects were on regular medication for cardiovascular diseases (hypertension and heart disease) and/or osteoarthritis of the knee joints and/or low back pain. The remaining 15 elderly individuals did not take regular medication. Moreover, prior to the study, we performed the mini-mental state examination, which all subjects passed (borderline passing score of 25). Experimental procedures were explained to subjects in advance, and their individual written consent was obtained. The present study was performed in accordance with the Declaration of Helsinki and approved by the Institutional Ethical Committee of Nagasaki University (No. 07051782).

### 2.2. Measurements

The tactile-pressure threshold of the distal palmar pad of the index finger was evaluated using Semmes–Weinstein monofilaments (North Coast Medical, Morgan Hill, CA, USA). We used 20 types of filaments ranging in weight between 0.004 and 447 g. The esthesiometer pressure in grams for each filament was converted to log_10_ 0.1 mg, yielding a scale composed of intervals of approximately equal intensity between filaments. Subjects were tested with their eyes closed after receiving clear instructions. The target area was marked on the volar side of the distal phalanx of the dominant index finger. Each filament was pushed into the target area until it bent by approximately 90° for one second. The threshold was recorded as the smallest filament diameter that was perceived in at least 80% of its applications (5 trials). SBF was monitored continuously on the palmar surface of the third finger of the dominant hand using a laser–Doppler flowmeter (ATBF-LC1, Unique Medical, Tokyo, Japan). SBF and the marking signal were simultaneously stored on a computer using an analog–digital converter (UAS-108S, Unique Medical, Tokyo, Japan) at a sampling frequency of 1 kHz and analyzed using computer software (Unique Acquisition 2.11, Unique Medical, Tokyo, Japan).

### 2.3. Braille Character Discrimination Task

We made flat plates with raised letters, as previously reported [10,11]. The convexities (diameter: 3 mm, height: 1 mm) of the plates were arranged according to the Braille code. Each flat plate was located on the back face of an opaque board to simulate blind conditions. Subjects were instructed to start touching one of the flat plates upon receiving our cue.

None of the subjects had ever attempted Braille reading. The Braille character discrimination task was performed in a soundproof room at an ambient temperature of 22–25 °C. After all, preparations were finished, each subject was allowed to sit on a chair for more than 5 min to stabilize their cardiovascular variables. The flat plate presented to subjects was randomly selected from 50 types of Braille codes corresponding to Japanese. Each subject was asked to touch a flat plate with raised letters for 15 s using the index finger of the dominant hand. After a rest for 30 s, each subject was asked to answer the Braille character that they touched by consulting a Braille chart.

### 2.4. Data Analysis

The difference in the tactile-pressure threshold due to age was examined using an unpaired *t*-test. The data of SBF were averaged every 1 sec. The mean value of SBF obtained for 15 s before touching the flat plates was defined as the baseline level. Changes in SBF were expressed as percent changes from baseline levels. These data were classified into two age groups (older group and young group) and were then averaged among subjects. The difference between the two age groups was statistically compared using a two-way analysis of variance (ANOVA). The mean change and peak response arrival time in SBF to Braille reading were measured, and differences between the two age groups were compared using an unpaired *t*-test. Older subjects were divided into two groups (hypertensive medication users and non-users), and comparisons of responses in SBF between these two groups were performed using the unpaired *t*-test. The significance of differences was defined as *p* < 0.05.

## 3. Results

### 3.1. Age-Related Changes in the Tactile-Pressure Threshold

**Figure 1:**
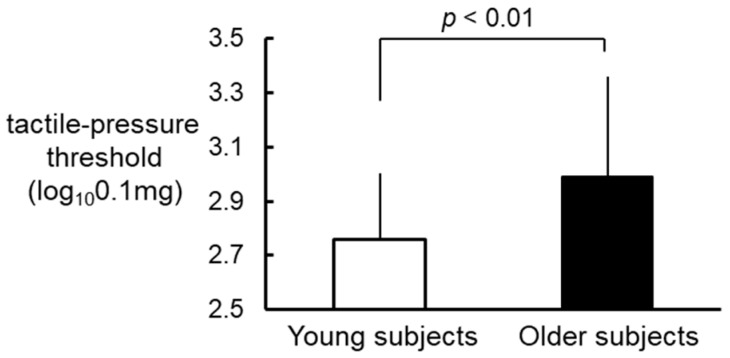
Comparison of the tactile-pressure threshold between young (open bar) and older (closed bar) subjects. Values are shown as the mean ± standard deviation.

The tactile-pressure threshold was higher in older subjects (2.99 ± 0.37 log_10_ 0.1 mg) than in young subjects (2.76 ± 0.24 log_10_ 0.1 mg) (Figure 1). The unpaired *t*-test revealed a significant difference in the tactile-pressure threshold between the two age groups (*p* < 0.01).

### 3.2. Time Courses of Changes in SBF during Braille Reading

**Figure 2:**
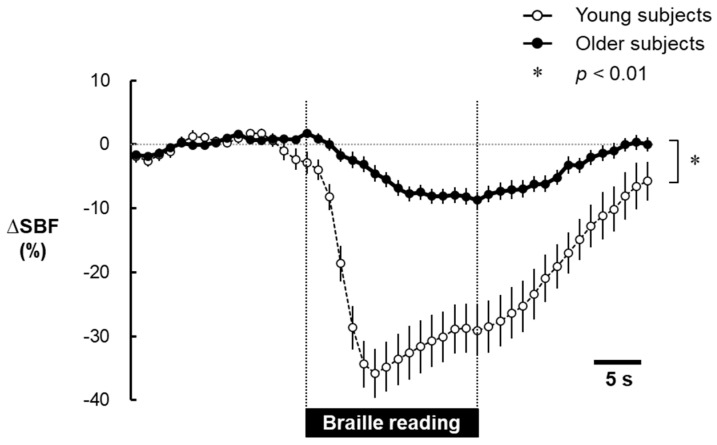
Time course of mean changes (∆) in skin blood flow in the finger (SBF) before, during, and after Braille reading in young (open circles) and older (solid circles) subjects. The duration of plate touching is shown by the horizontal bar. Values are shown as the mean ± standard error of the mean—percent changes from baseline levels. Significant difference between the two age groups: * *p* < 0.01.

The time courses of changes in SBF before, during, and after Braille reading are shown in Figure 2. The response of SBF to Braille reading markedly decreased in young subjects. The decrease in SBF reached a maximum value of −35.8 ± 20.2%, which was maintained during the later period of Braille reading. SBF returned slowly to the baseline level after the cessation of Braille reading. Moreover, SBF also decreased during Braille reading in older subjects (it decreased by −8.7 ± 8.9%). However, this decrease in SBF was markedly smaller in older subjects than in young subjects (*p* < 0.01).

### 3.3. Comparison of Mean Changes and Arrival Times of Peak Responses in SBF to Braille Reading

Mean changes in SBF to Braille reading in older and young subjects were −4.9 ± 7.0 and −25.8 ± 15.4%, respectively (Table 1). The SBF response was markedly smaller in older subjects than in young subjects (*p* < 0.01). Furthermore, decreases in SBF in older and young subjects peaked at 12.5 ± 3.1 and 8.8 ± 3.6 s, respectively, after the onset of Braille reading (Table 1). The decrease in SBF was delayed in older subjects than in young subjects (*p* < 0.01). On the other hand, the mean change in and arrival times of the peak response in SBF to Braille reading in antihypertensive medication users (*n* = 12) were −4.9 ± 6.8% and 11.9 ± 4.5 s, respectively, which did not significantly different from those in non-users (*n* = 25, mean change; −5.0 ± 7.3%, *p* = 0.96, arrival times of the peak response; 12.7 ± 2.3 s, *p* = 0.47).

## 4. Discussion

The aim of the present study was to identify age-related changes in the response of SBF induced by concentrated finger perception. A major novel result showed that the response of SBF to Braille reading was markedly smaller in older subjects than in young subjects. Furthermore, it appeared as a difference in the reaction time and was slower in older subjects. These results suggest that the regulation of finger blood flow associated with tactile sensation is affected by aging.

In the present study, 15 out of 37 elderly subjects had values higher than 2.83 log_10_ 0.1 mg (mean, 2.99 log_10_ 0.1 mg), which is generally considered to be normal for pressure detection [14]. This was consistent with that in previous studies [6,7] investigating the tactile-pressure threshold in healthy older individuals (mean, 3.02–3.21 log_10_ 0.1 mg). These results indicate that the sensory function of the hand is affected by aging and that normal values in the elderly need to be considered separately from those in adults. Regarding the decline in tactile sensation, morphological changes occur in peripheral receptors and the central nervous system due to aging (decreases in sensory receptors and brain weight) [15,16,17,18]. These changes are considered to contribute to the augmentation of hand sensation thresholds (attenuation of sensation and sensitivity) in the elderly.

SBF decreased during the Braille character discrimination task in the older and young groups. The glabrous skin areas of the hand are rich in arteriovenous anastomoses, which are innervated by sympathetic adrenergic vasoconstrictor nerves but lack the influence of active vasodilator nerves [19,20,21,22]. Previous studies reported that sympathetically mediated vasoconstriction was induced in glabrous skin by cold stress [19,20,23] and dynamic or isometric exercise [24,25,26]. Thus, sympathetically mediated vasoconstriction, induced by concentrated finger perception, contributes to reductions in finger SBF. On the other hand, aging is associated with several abnormalities in autonomic nervous system function that may impair adaptation to stress in the elderly. Previous studies reported that the response of skin sympathetic nerve activity to a cold stimulus was weaker in older subjects than in young subjects [27,28]. In the present study, the SBF response to Braille reading was markedly smaller and slower in older subjects than in young subjects. The attenuated sympathetic response due to aging may be attributed to a difference in the response of cutaneous vasoconstriction to Braille reading.

A correlation was previously reported between changes in finger blood flow and volume [11]. This finding suggested that the variability of SBF reflects changes in finger volume. In other words, a decrease in finger volume may induce a reduction in skin surface pressure, which, in turn, augments cutaneous tissue deformation. Fluctuations in finger blood flow may indirectly influence tactile sensitivity via the mechano-elastic characteristics of cutaneous tissue, which are produced by changes in finger volume. Therefore, the attenuating effect of aging on the response of SBF during Braille reading reflects differences related to the attenuation of finger volume changes. This attenuating effect on the cutaneous vasoconstrictor response may be related to the age-related decline in tactile sensibility. Moreover, the delay in the SBF response to Braille reading may reflect the delay in the perceptual judgment of finger sensations.

## 5. Conclusions

In conclusion, the tactile-pressure threshold of the finger was significantly increased by aging, whereas the response of finger SBF to concentrated finger perception was decreased and delayed by aging. Age-related changes in tactile sensibility may be related to alterations in the regulation of SBF.

## Figures and Tables

**Table 1:** Comparison of the mean change and peak response arrival time in SBF to Braille reading between age groups.

	Older Group (*n* = 37)	Young Group (*n* = 27)	*p*-Value
Mean response in SBF (Δ%)	−4.9 ± 7.0	−25.8 ± 15.4	<0.01
Peak response arrival time (s)	12.5 ± 3.1	8.8 ± 3.6	<0.01

Values are shown as the mean ± standard deviation.

## Data Availability

Data sharing not applicable.
